# Electricity, &c.

**Published:** 1889-06

**Authors:** 


Electricity and the methods of its employment in removing superfluous hair and other facial blemishes, by Plim S. Hays. M. D., late Professor of Chemistry and Toxicology, Woman's Medical College; Professor of Analytical Chemistry, Chicago College of Pharmacy; Professor of Gynecology, and of Electro Therapeutics, Chicago Policlinic etc. Chicago, W. T. Keener, 1889.

This book has been written, the author states, in answer to the many qnestions asked by brother physicians relative to the proper use of electricity in removing hairs, blemishes, etc.

